# Glucose Sensor Using Redox Active Oligonucleotide-Templated Silver Nanoclusters

**DOI:** 10.3390/nano9081065

**Published:** 2019-07-24

**Authors:** Kathryn L. Schroeder, Renee V. Goreham, Thomas Nann

**Affiliations:** 1 The MacDiarmid Institute, Victoria University of Wellington, Wellington 6012, New Zealand; 2 School of Chemical Physical Sciences, Victoria University of Wellington, Wellington 6012, New Zealand; 3 The School of Mathematical and Physical Sciences, University of Newcastle, Newcastle 2300, Australia

**Keywords:** nanocluster, silver, glucose, photoluminescence, DNA, oxidation, biosensing, reversible

## Abstract

Redox active, photoluminescent silver nanoclusters templated with oligonucleotides were developed for glucose sensing. The silver nanoclusters had a photoluminescent emission at 610 nm that reversibly changed to 530 nm upon oxidation. The reversible emission change was measured with photoluminescent spectroscopy and used to detect H_2_O_2_, which is a by-product of the reaction of glucose with glucose oxidase. The ratio of the un-oxidised emission peak (610 nm) and the oxidised analogue (530 nm) was used to measure glucose concentrations up to 20 mM, well within glucose levels found in blood. Also, the reversibility of this system enables the silver nanoclusters to be reused.

## 1. Introduction

Oligonucleotides can template silver ions (Ag^+^) that can be reduced to form silver nanoclusters (Ag NC) with tuneable size, and photoluminescence (PL) properties. Physical properties of the Ag NCs are directed by the specific nucleotide base sequence of the oligonucleotide and the affinity of Ag^+^ for DNA, particularly cytosine (C) bases [1,2,3,4,5,6,7,8,9]. The non-toxic nature and PL properties of Ag NCs make them suitable for applications in bioimaging or detection of biological molecules [1,2,3,4,5,6,7,9,10,11,12].

Glucose detection for managing diabetes is an example of a biological molecule detection system [13]. A glucose sensor should detect glucose levels up to 13 mM [14,15,16]. Glucose measurement techniques typically use an enzyme, such as glucose oxidase (GOx, also abbreviated to GOD), to produce H_2_O_2_. In electrochemical glucose sensing devices, H_2_O_2_ concentration is determined by measuring the O_2_ consumption, H_2_O_2_ production, or electron transfer from GOx to an electrode [17]. In other systems, the H_2_O_2_ oxidises a dye or nanomaterial to show a proportional change to the amount of glucose present [18,19]. Ag and Au nanoparticles/nanoclusters (NPs/NCs) used in this process have typically had irreversible changes, such as aggregation or etching [20]. This was seen with Ag NPs immobilised with GOx in a hydrogel. There was decreased absorbance with increasing glucose concentration, partly caused by H_2_O_2_ degrading the NPs [21,22]. Thiol capped Au NPs conjugated with GOx visibly changed colour. There was a linear correlation of glucose concentration with an absorbance red-shift for glucose concentrations between 0.056–0.56 mM. This was below the lower concentration present in human blood, but is appropriate for urine glucose concentrations, which are lower than in blood (1.4 mM is the normal limit) [23,24]. H_2_O_2_ has also been shown to break and reform citrate capped Ag NPs, which was observed by an absorbance shift [25]. Other PL based glucose sensors indicated glucose concentration by change in PL intensity when bind to glucose. An example of this is the continuous measurement device, Eversense, which has a polymer imobilised in a hydrogel that reversibly binds to glucose [26,27].

Ag NCs can be synthesised by incubating Ag^+^ with an oligonucleotide, followed by reduction producing photoluminescent NCs. It has been observed that some Ag NCs undergo a PL emission change on oxidation. This includes C-rich oligonucleotide templated Ag NCs [3,28,29], and glutathione capped Ag NCs (etching by H_2_O_2_ increased blue PL) [30]. The H_2_O_2_ detection limit for these Ag NCs was 0.025 µM, well below the glucose concentration in human blood [30]. Polymethacrylic acid and sodium salt (PMAA) and polyvinylpyrrolidone (PVP) templated, red-emissive Ag NCs acted as a turn-on detector for H_2_O_2_ and glucose, so that the PL was enhanced with increasing glucose concentration in the range of 0.01–0.5 mM, attributed to NC etching [31].

This paper reports a method to detect glucose by monitoring the emission wavelength of Ag NCs. First, Ag NCs were synthesised by templating with oligonucleotides and reducing with sodium borohydride (NaBH_4_). They were characterised by PL spectroscopy, matrix assisted laser desorption ionisation mass spectroscopy (MALDI-MS), and circular dichroism (CD). In the presence of H_2_O_2_, a by-product of the reaction between glucose oxidase and glucose, it was found that these Ag NCs reversibly oxidised in the presence of H_2_O_2_, dependent on glucose concentration. The results were compared to the values given by a commercial electrochemical sensor. This process improved on other NP/NC glucose sensing systems because the PL change was reversible and glucose could be detected within a concentration range of 0–10 mM, the normal range for humans.

## 2. Materials and Methods

### 2.1. Materials

Silver nitrate (AgNO_3_, Sigma Aldrich, Castle Hill, Australia), oligonucleotide sequence 5’ -ACC TCA GTG TGTC CCC CCC CCC CCC -3’ (IDT or Alpha DNA, with standard desalting), sodium borohydride (NaBH_4_, Sigma Aldrich), Milli-Q water, glucose, glucose oxidase from *Aspergillus niger* (Type II, ≥15,000 units·g^−1^, Sigma Aldrich), 50 mM sodium acetate (NaOAc) buffer (pH 5.4, Sigma Aldrich).

### 2.2. Silver Nanocluster Synthesis

Ag NCs were synthesised similarly to previously published procedures, with a 6:1:6 ratio of aqueous solutions of Ag^+^:oligonucleotide:NaBH_4_ [3,6,10,11,32]. Briefly, 100 µM AgNO_3_ was combined with 100 µM oligonucleotide and vortexed for 1 min, followed by incubation (10 min, dark, room temperature). Freshly made 100 µM NaBH_4_ was added dropwise to the silver oligonucleotide solution while vortexing (1 min) and incubated for 10 min in the dark by which time photoluminescence under UV light had developed. Finally, samples were centrifuge filtered using Amicon 3 kDa MWCO centrifugal filters (Centricon, Castle Hill, Australia) according to the manufacturer’s protocol and the purified Ag NCs were stored at 4 °C.

### 2.3. Characterisation

Initial PL measurements of oxidised and un-oxidised Ag NCs were done using an Edinburgh F980 fluorescence spectrophotometer (Edinburgh, UK). Quantum yield (QY) measurements were done using an integrating sphere. MALDI-MS spectra were measured using 3-hydroxypropionic acid (3-HPA) and a combination of anthranilic and nicotinic acids as matrices because they gave clear spectra. CD spectra were measured in an Applied Photophysics Chirascan-plus spectrometer (Surrey, UK).

### 2.4. Protocol for Glucose Sensing

The procedure was adapted from Zong et al. for glucose sensing with Ag NPs [20]. Glucose (0–20 mM; 50 µL) and GOx (200 µL, 16.3314 mg·mL^−1^, 15 000 U·g^−1^, in pH 5.42, 50 mM NaOAc) were combined in a 96-well plate and incubated (~50 min, RT) to allow H_2_O_2_ production without Ag NCs because Ag^+^ is known to interfere with GOx activity [33]. Freshly prepared Ag NCs (50 µL) were added and mixed by pipetting. PL and absorbance were measured using a plate reader at 10 min intervals with shaking in between measurements for continual reactant mixing. PL wavelengths measured were λ_Ex_ 555 nm, λ_Em_ 612 nm (unoxidised peak), and λ_Ex_ 445 nm, λ_Em_ 530 nm, (oxidised peak). Absorbance wavelengths measured were 424 nm and 553 nm. The ratios of the PL or absorbance intensities at the two wavelengths were plotted against glucose concentration to produce a calibration plot for use in determining unknown glucose concentrations (standard deviation calculation is shown in Equations S1 and S2). Measurements for glucose sensing tests were done with a plate reader (Perkin Elmer EnSpire^TM^ 2300 Multilabel Reader, Waltham, MA, USA) to increase measurement speed. The wavelengths used were determined with fluorescence/UV-Vis spectrophotometers and optimised by the plate reader. This process was repeated with phosphate buffer saline (PBS) (instead of water) spiked with glucose.

To detect glucose in blood, blood samples were taken by a nurse into grey glucose tubes. The blood was allowed to coagulate (35 min, R.T.) and centrifuged (15 min, 4 °C, 2000 × g). The separated serum was removed into polypropylene vials. The same procedure used to measure known glucose concentrations was used to measure blood glucose concentration, with blood serum (50 µL) used instead of the glucose (50 µL). Known glucose concentrations were also measured to be used as a calibration plot.

## 3. Results and Discussion

**Figure 1:**
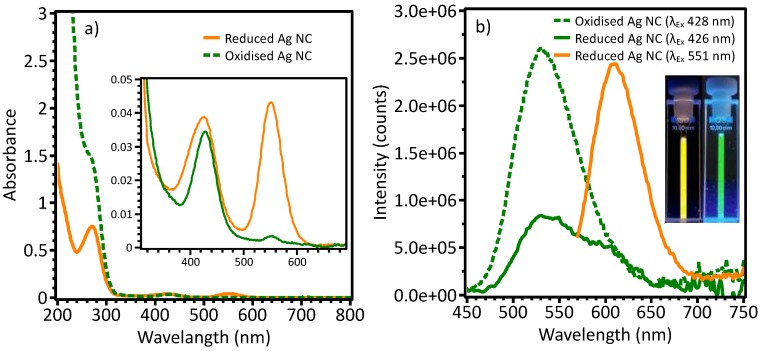
(**a**) UV-visible absorption spectra of un-oxidised (orange solid line) and oxidised (green dashed line) Ag NCs. (**b**) Emission spectra of un-oxidised (solid lines) and oxidised (dashed line) Ag NCs. Inset: Fresh, un-oxidised Ag NC had bright orange-yellow PL, and Ag NC after oxidation with H_2_O_2_ had green PL.

Oligonucleotide stabilised Ag NCs were synthesised following previously reported methods using a new oligonucleotide sequence [3,6,10,11,32]. Many oligonucleotide sequences were trialed but the one used in this work had the highest QY, stability and largest photoluminescent emission change. Silver ions were added to the oligonucleotide and subsequently reduced producing photoluminescent Ag NCs. Changes in the CD spectrum (Figure S1) indicated that the oligonucleotides had changed conformation, evidence of NC formation. The Ag NCs showed strong PL intensity and good stability against oxidation during storage. The Ag NCs had a strong emission peak at 610 nm and a weaker emission peak at 530 nm (Figure 1b), and a QY of 58 ± 7% at an excitation wavelength of 525 nm, corresponding to the absorbance at 550 nm (Figure 1a). Exciting the un-oxidised Ag NCs at 426 nm excited the species with the 530 nm emission, and also the 610 nm emitting species to a lesser extent to the 551 nm excitation. Ag NC oxidation (chemically with H_2_O_2_, or with glucose and glucose oxidase), caused an increase in the 530 nm emission peak resulting in green photoluminescent Ag NCs. Corresponding to the appearance of green emission with oxidation was a loss of the 530 nm peak in the UV-Vis absorbance spectrum while the other peaks remained (Figure 1). The absorption peak at 270 nm is characteristic for DNA absorption [34]. Addition of NaBH_4_ to the oxidised Ag NCs caused the original UV-Vis absorption and PL characteristics to return (Figures S2 and S3).

The Ag NCs had high intensity PL emission (610 nm) that was stable for more than two weeks stored at 4 °C. After this time, the AgNCs would oxidise (PL emission 530 nm) but could easily be reduced to form the original Ag NC again for use. Oxidation by H_2_O_2_ at concentrations analogous to those produced by biological concentrations of glucose (3–5 mM) caused the PL emission peak at 530 nm to increase. These two emission wavelengths are common for Ag NCs and have been associated with Ag NC size, so that oxidative PL change would imply size change [35]. However, the findings of this study suggest that these two peaks might be caused by different oxidation states, with no size change. A number of groups who used NC/NPs as glucose sensors saw an irreversible PL/absorbance shift or quenching caused by NC/NPs agglomeration or disintegration [20,23,30,31]. In contrast, the Ag NCs in this study had a reversible PL change, which is advantageous for use as a cheaper, reusable sensor. The mechanism for this reversible oxidative PL change is still unknown. Possible explanations are conversion of Ag atoms to Ag^+^, or change in the way that the oligonucleotide and NCs interact [8,36]. Another study showed reversible PL quenching via a similar mechanism but the PL emission quenched upon H_2_O_2_ exposure [29]. The presence of the green emission in the un-oxidised sample spectrum indicates that the species causing the emission at 530 nm is present in both oxidised and un-oxidised samples. The oxidised nanocluster had much lower intensity emission than the un-oxidised nanocluster (about an order of magnitude). Two species of Ag NCs hypothesised to be present, with oxidation, causing the primary species to change, producing an NC that emits at 530 nm. Synthesis with deoxygenated reagent solutions would more fully reduce Ag NCs as they are synthesised. Alternatively, both species may be present in a fully reduced sample, with the 530 nm emitting species being less emissive than the 610 nm emitting species. Ag NCs with a PL change from long to short wavelengths all had a sequence with a C-only section [28,30]. The PL wavelength and intensity of each Ag NC would have been affected by the length of the C-only sections and the other bases present.

**Figure 2:**
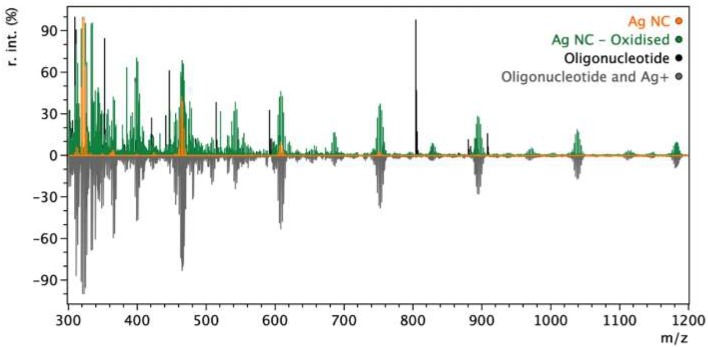
MALDI-MS of the oligonucleotide (black), oligonucleotide with AgNO_3_ (grey, “Oligonucleotide and Ag^+^”), Ag NC after reduction (orange) and the oxidized Ag NC (green, “Ag NC—Oxidised). The spectrum of oligonucleotide and is shown inverted for easier comparison with the other data. Individual spectra and peaks are shown in Figures S4–S7, and Table S1.

MALDI-MS was conducted on the oligonucleotide, oligonucleotide with AgNO_3_, the reduced Ag NCs and the oxidised Ag NCs (Figure 2). As shown, once the AgNO_3_ is added to the oligonucleotide it fragments differently. This is not surprising as silver and oligonucleotide complexing has been well studied. Once the Ag NC is formed via reduction with sodium borohydride, the peak intensity decreases. Once an oxidant is added, the MS is similar to both the unreduced (AgNO_3_ and oligonucleotide) and Ag NCs spectrum. This give further evidence that photoluminescent change is from an oxidation process, which leads to the formation of a more dominant and smaller cluster (4–5 Ag atoms). Evidence of this is given elsewhere, for example Copp et al. showed that two different sized clusters produced two photoluminescent emissions [35]. Recently, Huard et al. was able to define the crystal structure of a DNA stabilised Ag NC, showing an entire 8 atom unit cluster with a shape reminiscent of the “Big Dipper” asterism [37]. This gives evidence that shape constraints could occur inducing oxidation of certain types DNA Ag NCs. This could lead to the production of a more dominant and smaller Ag NC exhibiting a different photoluminescent emission.

**Figure 3:**
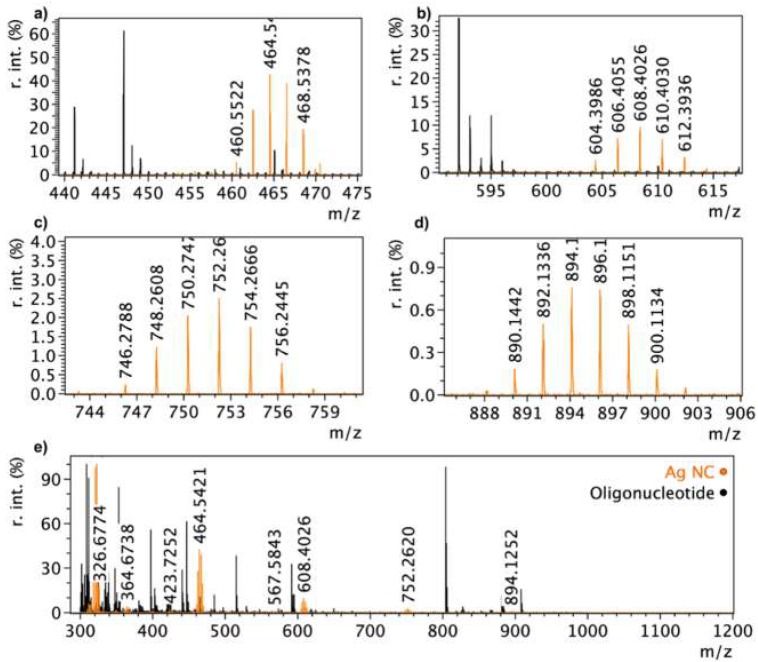
Corresponding MALDI-MS spectra for oligonucleotide on its own and Ag NCs showing 3–8 Ag atom Ag NCs (**e**) and zoomed regions of peaks of interest (**a**–**d**). The oligonucleotide on its own shows sets of three peaks in descending intensity, The Ag NC spectrum (orange peaks) contains distributions of peaks to Ag isotopes, and is missing the peaks associated with the oligonucleotide (black peaks).

Ag NCs contained 3–8 atoms, shown by MALDI-MS and determined by comparing peaks to Ag isotope distributions [38]. At 465 and 894 m/z (Figure 3a,d), there were distributions of six peaks (with two centre peaks), indicating Ag_5_ NCs. Seven peak distributions (with one centre peak) at 752 and 608 m/z (Figure 3b,c) indicated Ag_6_ NCs. These Ag NC peaks were distinct from the oligonucleotide peaks present in the spectra of the oligonucleotide on its own, which begin with a tall peak followed by at least two peaks descending in height (Figure 3e). The MALDI spectrum for the Ag NC shows a distant change compared to the oligonucleotide on its own. When oxidized, the spectrum changes to show peaks present in both the oligonucleotide and the Ag NC, indicating a change in cluster structure. Transition electron microscopy (TEM) images were not acquired because at the size of these Ag NCs. Typical TEM images do not show the true size of the Ag NCs, rather showing aggregates that are formed during measurement [39,40,41].

The Ag NCs used in this study have strong PL that can be reversibly oxidised. Oxidation changes their observed PL wavelengths rather than bleach them. These characteristics make them ideal for the detection of an oxidation process, such as in glucose sensing.

**Figure 4:**
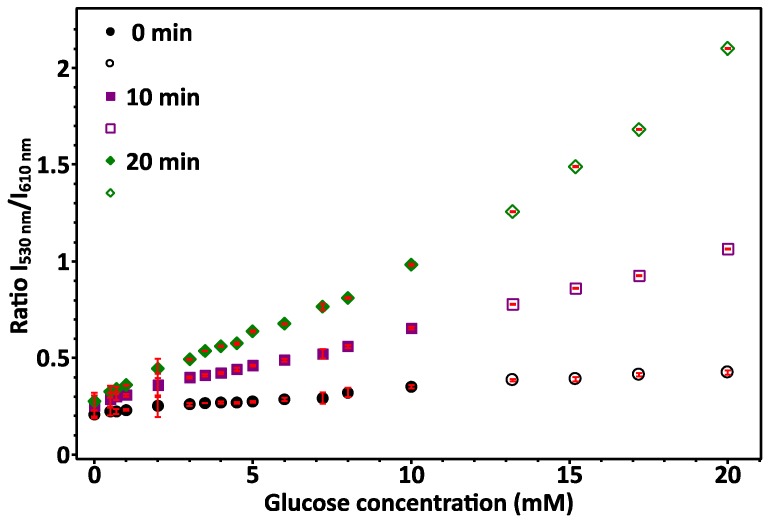
Glucose sensing measuring change in PL emission of the two wavelengths. Measurements were done at λ_Ex_ 555 nm, λ_Em_ 612 nm (unoxidized peak), and λ_Ex_ 445 nm, λ_Em_ 530 nm (oxidised peak). Ratios of the mean (three replicates) 610 nm (un-oxidised) and 530 nm (oxidised) emission intensities corresponded to glucose concentrations. Data was measured at ~10 min intervals. A linear range was seen for glucose concentrations between 0 and 10 mM. Error bars are standard deviation.

Initial combination of GOx with different glucose concentrations (0–20 mM) changed the GOx from yellow to colourless with higher glucose concentration. Glucose and GOx produced concentration dependent amounts of H_2_O_2_, which oxidised the Ag NCs, (greater change with higher glucose concentration). Illumination with UV light, showed that samples with lower glucose concentration had orange (610 nm) PL, changing to yellow (a mixture of 610 and 530 nm emission), and then green (530 nm emission) as glucose concentration was increased. This supports the hypothesis that there are two Ag NC sizes producing two separate emissions. In addition, the production of H_2_O_2_ decreases the overall pH when the gluconic acid is formed, which may aid in the changing structure of the Ag NC. This was reflected in the PL measurements (Figure S1 is in water), which showed a clear trend of the 610 nm (red) emission intensity decreased, while the 530 nm (green) emission intensity increased with increasing glucose concentration. The ratios of the mean unoxidized and oxidised intensity values for each glucose concentration were plotted against glucose concentration to get a calibration plot (Figure 4). This same procedure was done alongside measurements for samples with unknown glucose concentrations. PL emission intensity measurements were repeated at 10 min intervals to investigate how the emission intensities changed over time. The results followed a similar trend, while the ratio between the two emissions decreased, so measurement of unknown glucose concentrations should be done at the same time as the calibration solutions. The increased rate of oxidation with time could be attributed to oxygen in the air (since the well-plates were not sealed or under vacuum), or from the solvent (water) and also the change in pH of the solution. As pH can strongly effect the stability of the DNA secondary structure, this may increase the rate of oxidation over time. It would not be due to the H_2_O_2_ because it would be used up within 10 min of addition, making it important to measure PL upon Ag NC addition. A linear region between 0 – 10 mM could then be used as the range within which to measure glucose. The lower detection limit was not found as it was not relevant to this study, although it may be useful for other oxidation detection applications. Since the PL change caused by Ag NC oxidation is reversible via reduction, the Ag NCs can be reused for multiple measurements.

Measurements were done using glucose solutions made in PBS to simulate similar conditions to biological fluids (Figure S5). The response was similar to measurements using glucose in water. It was assumed that GOx was specific for glucose over other blood components due to measurements by Zong et al. that had already shown this specificity [20].

When measuring unknown glucose concentrations in blood, calibration solutions were made in water as done previously (Figure S8). Blood serum samples were measured in triplicate with a similar concentration of GOx and Ag NCs as the calibration solutions (after centrifuge filtration and concentration, the Ag NC solutions were re-diluted to 4 mL). The ratio of the emission intensities was used to find the unknown glucose concentration of the blood serum samples. These were compared to values from a commercial meter (CareSens Dual). The experimental blood glucose level was similar to the value determined by the commercial sensor. Another sample was further from the commercially determined value. This discrepancy was likely caused by other components in the blood serum interfering with the oxidation process of Ag NCs. This was observed visually—the wells containing blood serum appeared to be greener than the calibration wells that had comparable glucose concentrations. The other oxidising components need to be accounted for when doing blood glucose measurements. To be commercially viable, the effect of blood serum in oxidising the Ag NCs would ned to be taken into account, and a simpler method for re-reducing the Ag NCs re-set the system for the next measurement would be required. This could possibly be done in a device using an electrical current.

## 4. Conclusions

Silver nanoclusters made with oligonucleotides were used to measure glucose by measuring changes in PL emissions [13]. These NCs contained approximately 5–6 silver atoms, and had a PL maximum of 610 nm in their un-oxidised state, and a PL emission maximum of 530 nm in their oxidised state. H_2_O_2_ produced by the reaction of glucose with glucose oxidase (GOx) causes oxidation of the Ag NCs that was reflected in a PL emission change from red to green with increasing glucose concentration. The glucose concentration of an unknown sample can be determined by comparing the ratios of un-oxidised:oxidised emission intensity with samples of known glucose concentration. The reversibility of this oxidative PL change allows this to be a reusable sensor. To use this system to determine blood glucose, the components in blood serum that also cause Ag NC oxidation need to be accounted for. Having been shown to be able to be used to detect oxidation in the context of glucose sensing, these Ag NCs could also be used to detect oxidation in other ex vivo, in vitro, and in vivo contexts.

## Supplementary Materials

The following are available online at https://www.mdpi.com/2079-4991/9/8/1065/s1, Figure S1: CD spectra of Ag NCs, Figure S2: UV/vis absorption spectra of Ag NCs, Figure S3: Excitation spectra of Ag NCs, Figures S4–S7: MALDI-MS spectra, Table S1: MALDI-MS peaks, Figure S8: Calibration curves.
