# Supplementary material for: Glucose Sensor Using Redox Active Oligonucleotide-Templated Silver Nanoclusters
Source: Nanomaterials (Basel). 2019 Jul 24;9(8):1065. doi: 10.3390/nano9081065 (PMC6722757; doi:10.3390/nano9081065)
Supplement: Supplementary file 1 [file nanomaterials-09-01065-s001.pdf]

Supplementary

Instrumentation and calculations

Absorption

UV-Vis absorption spectra were obtained with a quartz cuvette in a Cary® Bio-50 spectrometer equipped with a Xenon flash lamp. Samples were scanned from 200–800 nm with a 0.1 s dwell time and background-subtracted with a water blank.

Emission

Photoluminescent data was obtained using a quartz cuvette in an Edinburgh Instruments FLS980 spectrophotometer equipped with a continuous Xenon lamp, single-grating excitation and emission monochrometers, and a Hamamatsu R928P thermoelectrically-cooled photomultiplier tube detector. Typical emission spectra resulted from 5 scans of 0.2 s dwell time each, using moderate excitation ($\Delta\lambda$ = 1–4 nm) and fine emission ($\Delta\lambda$ < 0.5 nm) bandwidths, optimising peak signal to ~30,000 counts per second (cps) with sample optical density of <0.1 au at the excitation wavelength.

Integrating sphere measurements were performed with a direct excitation geometry and 5 scans of 0.2 s dwell time each, using broad excitation ($\Delta\lambda$ = 10–12 nm) and fine emission ($\Delta\lambda$ < 0.5 nm) bandwidths, optimising detected excitation peak signal to ~750,000 cps with sample optical density <0.1 au at the excitation wavelength. Bandwidths and emission intensity were preserved between sample and blank measurements for accurate quantum yield calculations. Quantum yields were calculated using an integrating sphere and correcting the observed QY for self-absorption effects with a standard 90°, 1 cm path length, <0.1 au OD emission scan (PL90°).

MALDI-MS

Matrix (10 mg/mL) was dissolved in a 50% acetonitrile, 0.1% trifluoro-acetic acid (TFA) solution. Sample (oligonucleotide or Ag NC, 10 µL) was combined with matrix (10 µL), and spotted (1 µL) onto the plate. This solution was double diluted with matrix solution (10 µL) and water (10 µL) and spotted onto plate (1 µL). This was repeated a third time, followed by drying the spots. Negative reflector mode was used to measure smaller masses (≤5 kDa).

Results

Characterisation

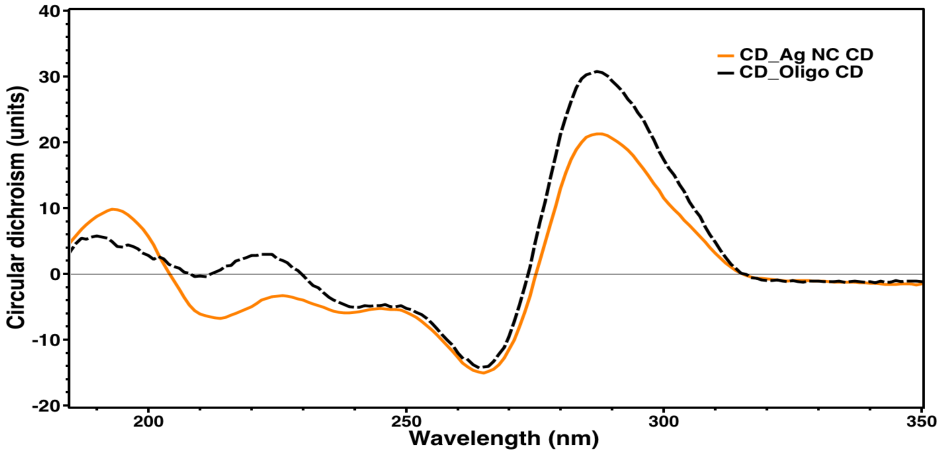

Figure S3. Circular dichroism of Ag NCs, and the templating oligonucleotide on its own. There was some conformational change with addition of silver, additional more evidence that Ag NC formation was associated with the oligonucleotide.

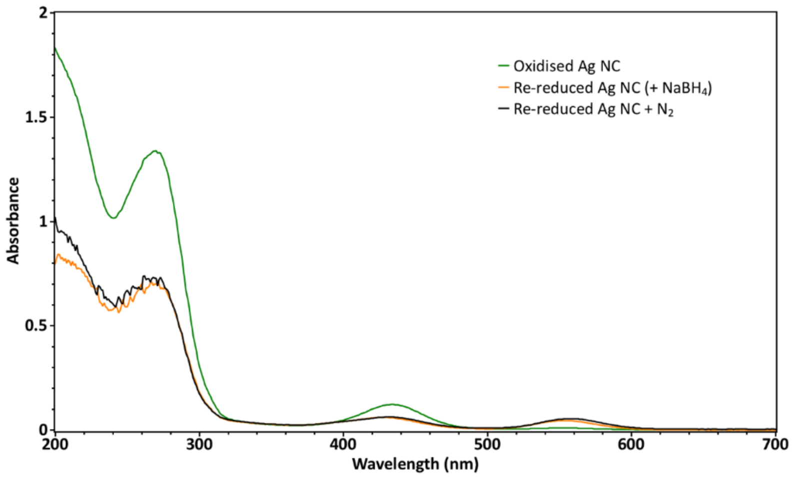

Figure S1. UV-visible absorption spectra of an ageing Ag NC sample that had oxidised with time (green curve) was re-reduced with $NaBH_4$ this caused the return of the 560 nm absorbance peak (orange and black curves). Bubbling N_2 gas into the re-reduced sample to remove oxygen gave a slight increase in the 560 nm peak. Removing oxygen likely have a small effect on the amount of Ag NC oxidation. Making Ag NCs with deoxygenated water/reagent solutions and bubbled with N_2 may cause the 410 nm peak to completely disappear, or for the 560 nm peak to increase further. The peak height difference between the oxidised and reduced (un-oxidised) Ag NCs is likely due to concentration change during reduction.

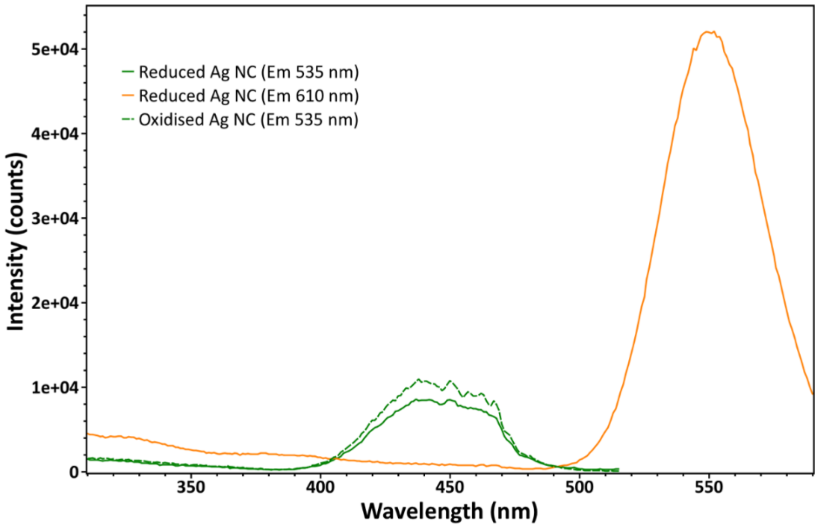

Figure S2. Excitation spectra show that the excitation peaks mostly match up with the absorption peaks. The peaks for the oxidised species in the reduced (un-oxidised) and oxidised Ag NCs match with each other, but are right-shifted (to 440 nm) compared to the corresponding absorbance peaks (425 nm). There is little evidence of other excitation peaks given that the spectra below 500 nm (for the reduced/un-oxidised emission) and 400 nm (for the oxidised emission) are flat relative to the peaks. The peak at 305 nm in the reduced (un-oxidised) spectrum is some sort of second order diffraction (half of 610 nm).

Mass Spectrometry

Mass spectra shown were acquired in negative reflector mode, and were measured in an m/z range of 300–1200. Major peaks are labelled in the spectra and further detailed in the table below.

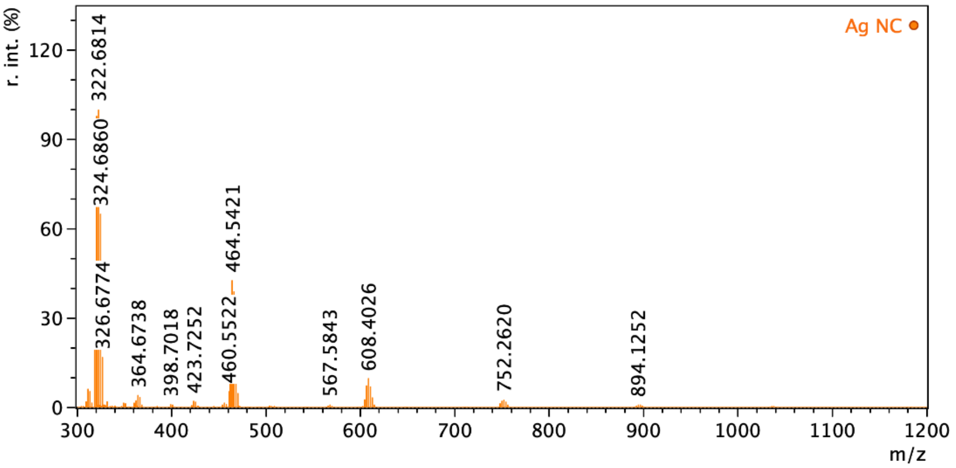

Figure S4. MALDI-MS of the Ag NC as synthesised normally, without any further treatment.

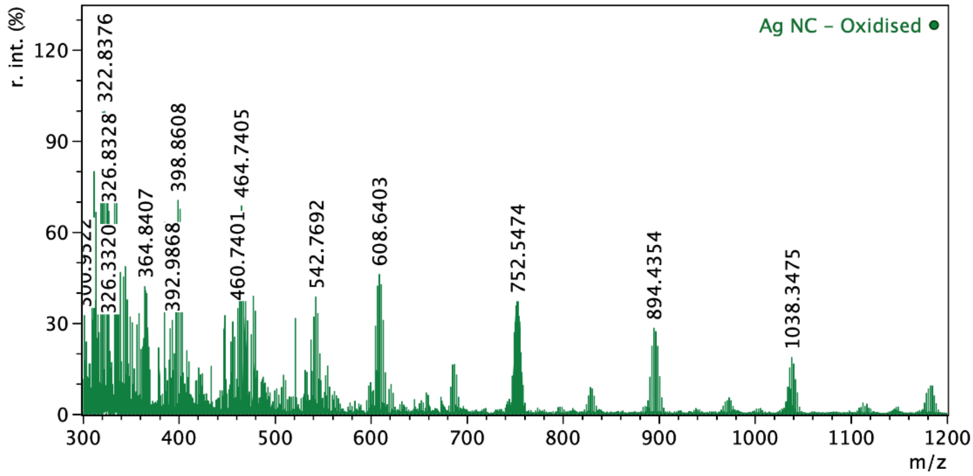

Figure S5. MALDI-MS of the Ag NC as synthesised normally, and then oxidised.

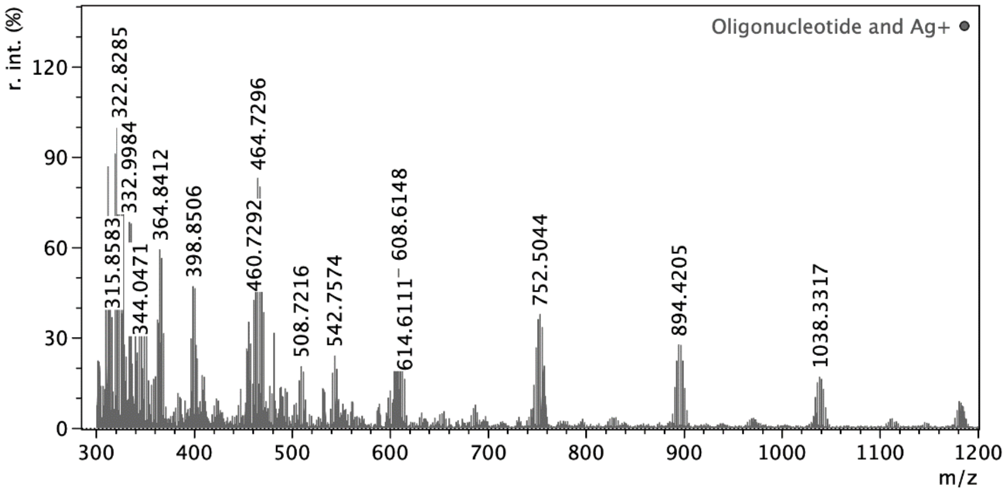

Figure S6. MALDI-MS of the oligonucleotide with Ag^+ without reduction.

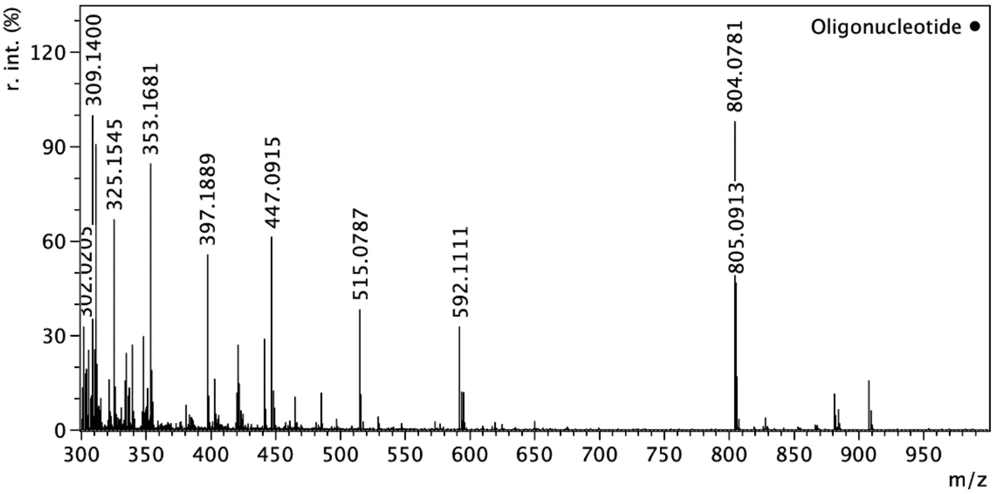

Figure S7. MALDI-MS of the oligonucleotide on its own.

Table S1. MS peaks for the oligonucleotide, oligonucleotide + Ag^+, Ag NC, and oxidised Ag NC, along with the expected masses of Ag NCs with different numbers of atoms. m/z is the mass to charge ratio, and r.int/ is the relative intensity of the peak. Coloured section are sets of peaks that appear typical of Ag NC isotopes peaks. Sets in bold match up with expected masses for Ag NCs. It looks like there are Ag_3 and Ag_7 NCs present.

**Oligonucleotide**		**Ag^+ and Oligonucleotide**		**Ag NC**		**Ag NC oxidised**		**Ag NC**	**Mw**
**m/z**	**r.int.**	**m/z**	**r.int.**	**m/z**	**r.int.**	**m/z**	**r.int.**		
301.01	13.44	309.85	45.79	**318.69**	**49.09**	309.87	50.21	Ag_3	323.7
302.02	32.84	311.27	86.96	**320.68**	**97.83**	311.30	80.13	Ag_4	431.6
303.03	17.93	311.86	71.02	**322.68**	**100**	311.86	74.27	Ag_5	539.5
304.02	19.45	312.29	42.27	**324.69**	**64.89**	313.87	66.58	Ag_6	647.4
306.02	25.39	313.85	64.27	326.68	16.84	**318.84**	**91.43**	Ag_7	755.3
307.97	10.98	315.86	36.80	361.83	2.19	318.99	30.00	Ag_8	863.2
309.14	100	**318.84**	**91.05**	364.67	3.97	**320.84**	**99.80**	Ag_9	971.1
310.14	25.49	**320.84**	**99.74**	366.67	3.45	**322.84**	**100**	Ag_{10}	1079
311.01	13.82	**322.83**	**100**	423.73	2.09	**324.83**	**95.41**		
311.14	90.74	**324.82**	**95.45**	425.72	1.71	325.32	70.87		
312.14	20.92	325.30	80.40	460.55	5.33	326.83	66.94		
315.00	10.07	**326.82**	**71.55**	462.55	27.57	333.01	95.10		
321.18	15.97	333.00	68.46	464.54	42.68	334.01	40.99		
325.15	66.90	335.00	67.86	466.54	38.87	334.99	95.74		
326.15	13.65	339.31	60.41	468.54	19.46	336.02	37.82		
333.97	15.61	362.82	35.94	470.54	4.70	336.94	38.81		
334.97	24.38	364.84	59.37	567.58	0.85	339.35	46.69		
335.97	10.78	366.84	56.43	604.40	2.59	342.06	45.30		
336.97	10.10	368.82	31.49	606.41	7.20	344.05	48.65		
337.17	13.35	396.85	29.78	608.40	9.65	364.84	41.98		
339.16	26.95	398.85	47.09	610.40	6.99	366.85	39.75		
348.00	29.74	400.85	46.38	612.39	3.21	385.08	63.28		
350.99	13.24	402.85	23.09	614.40	0.79	396.86	52.66		
353.17	84.55	453.74	26.44	**748.26**	**1.22**	398.86	70.36		
354.17	18.88	455.76	35.35	**750.27**	**2.05**	400.86	67.52		
397.19	55.74	457.75	28.01	**752.26**	**2.50**	401.06	42.12		
398.19	10.76	460.73	42.53	**754.27**	**1.75**	402.86	44.42		
403.05	16.20	462.72	73.67	**756.24**	**0.81**	460.74	34.70		
420.04	11.75	464.73	83.02	894.13	0.76	462.74	60.16		
421.05	27.02	466.72	80.18			464.74	68.69		
422.06	14.65	468.72	65.37			466.74	66.12		
423.04	6.11	470.72	38.55			468.74	54.07		
424.13	3.37	506.73	15.87			470.74	30.79		
425.04	5.03	508.72	20.55			476.92	38.87		
425.22	4.23	510.71	18.60			478.92	34.02		
441.20	28.83	**540.75**	**18.34**			537.06	24.47		
442.20	6.68	**542.76**	**24.08**			**540.78**	**32.03**		
447.09	61.29	**544.76**	**19.64**			**542.77**	**38.69**		
448.08	12.38	604.62	32.09			**544.78**	**33.26**		
449.10	6.99	606.62	48.60			**546.77**	**19.90**		
515.08	38.33	608.61	52.91			604.64	29.02		
516.07	11.27	610.61	47.68			606.64	42.28		
592.11	32.71	612.62	34.79			608.64	46.27		
593.12	12.03	614.61	16.35			610.64	42.74		

594.12	3.06	**748.52**	**26.73**	612.64	31.04
595.03	12.01	**750.52**	**36.11**	**748.55**	**26.98**
804.08	98.02	**752.50**	**37.72**	**750.55**	**35.84**
805.09	46.77	**754.52**	**33.36**	**752.55**	**37.22**
806.09	16.97	**756.52**	**20.58**	**754.54**	**32.37**
807.09	3.36	892.43	22.31	**756.54**	**20.30**
		894.42	27.64	892.46	22.50
		896.43	27.55	894.44	28.36
		898.44	22.28	896.46	27.26
		1036.32	15.14	898.44	22.49
		1038.33	17.02	1038.35	18.67
		1040.32	16.26		

Glucose Sensing

To calculate the standard deviation, the following equation was used:

$$x = \frac{I_{530} \pm \sigma_{I_{530}}}{I_{610} \pm \sigma_{I_{610}}} \quad \text{(S1)}$$

so

$$\sigma_x = \left(\sqrt{\left(\frac{\sigma_{I_{530}}}{I_{530}}\right)^2 + \left(\frac{\sigma_{I_{610}}}{I_{610}}\right)^2} \right) \times \left(\frac{I_{530}}{I_{610}}\right) \quad \text{(S2)}$$

where σ is the standard deviation, and I is the PL intensity.

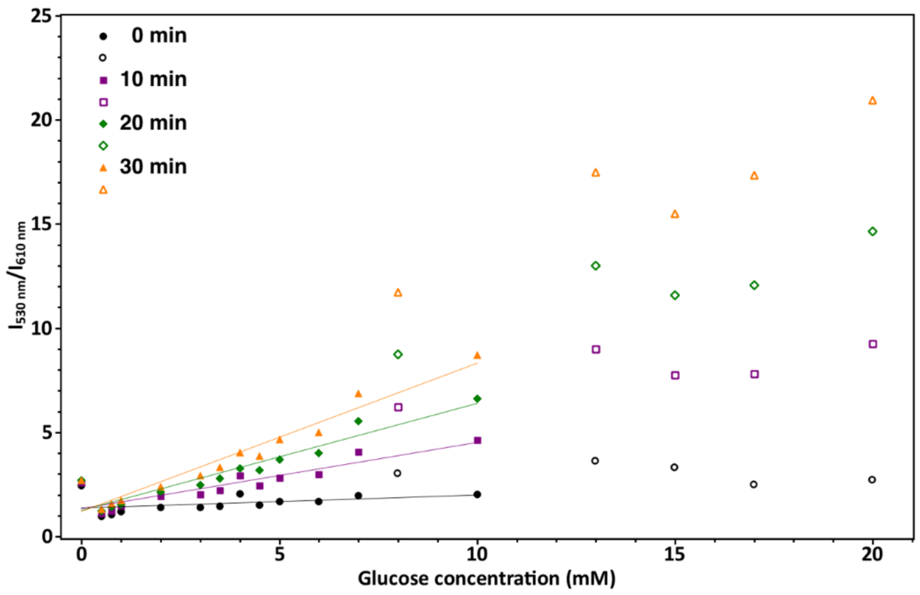

Figure S8. Calibration curves of glucose in PBS, measured at 10 min intervals.
